# Is in utero exposure to maternal socioeconomic disadvantage related to offspring ovarian reserve in adulthood?

**DOI:** 10.1186/s40695-018-0033-2

**Published:** 2018-03-16

**Authors:** Maria E. Bleil, Paul English, Jhaqueline Valle, Nancy F. Woods, Kyle D. Crowder, Steven E. Gregorich, Marcelle I. Cedars

**Affiliations:** 1 Department of Family and Child Nursing, University of Washington, Box 357262, Seattle, WA 98195 USA; 2 California Department of Public Health, California Environmental Health Tracking Program, Richmond, CA 94804 USA; 3 Department of Biobehavioral Nursing and Health Informatics, University of Washington, Seattle, WA 98195 USA; 4 Department of Sociology, University of Washington, Seattle, WA 98195 USA; 5 Department of Medicine, University of California San Francisco, San Francisco, CA 94143 USA; 6 Department of Obstetrics, Gynecology, & Reproductive Sciences, University of California San Francisco, San Francisco, CA 94143 USA

**Keywords:** Ovarian reserve, Ovarian aging, Menopause, Antral follicle count (AFC), Antimullerian hormone (AMH), Socioeconomic status (SES), Poverty, Neighborhood

## Abstract

**Background:**

Because the ovarian follicle pool is established in utero, adverse exposures during this period may be especially impactful on the size and health of the initial follicle endowment, potentially shaping trajectories of ovarian follicle loss and the eventual onset of menopause. Building on a robust literature linking socioeconomic status (SES) and menopausal timing, the current study examined adverse prenatal exposures related to maternal SES, hypothesizing that greater maternal socioeconomic disadvantage would be associated with lower ovarian reserve in the adult offspring.

**Methods:**

In a healthy, community-based sub-sample (*n* = 350) of reproductive age participants in the OVA Study (2006–2011), prenatal maternal SES was examined in relation to two biomarkers of ovarian reserve, antimullerian hormone (AMH) and antral follicle count (AFC). Prenatal maternal SES was assessed indirectly using maternal addresses abstracted from participant birth certificates, geocoded, and linked to US Census-derived variables, including neighborhood-level characteristics: education (% of individuals with a HS diploma); poverty (% of families below the poverty line); unemployment (% of individuals > 16 years who are unemployed); and income (median family income).

**Results:**

In separate covariate-adjusted linear regression models (following the backward elimination of main effects with *P* > .10), greater maternal neighborhood education was related to higher ovarian reserve as marked by higher levels of offspring AMH (beta = .142, *P* < .001) and AFC (beta = .092, *P* < .10) with models accounting for 19.6% and 21.5% of the variance in AMH and AFC, respectively. In addition, greater maternal neighborhood poverty was related to lower ovarian reserve as marked by lower offspring AMH (beta = −.144, *P* < .01), with the model accounting for 19.5% of the variance in AMH.

**Conclusions:**

Maternal socioeconomic disadvantage measured indirectly at the neighborhood level was associated with lower ovarian reserve among the adult offspring, independently of offspring SES and other potential confounding factors. This suggests SES-related adversity exposures may have a detrimental impact on the size or health of the initial follicle endowment, leading to accelerated follicle loss over time.

## Background

Younger age at menopause has been associated with an increase in cardiovascular risk for outcomes including ischemic heart disease, stroke, atherosclerosis, and cardiac-specific mortality which together account for a significant proportion of morbidity and mortality among women in the postmenopausal period [1–12]. The study of menopausal timing is limited, however, as menopause by definition is determined retrospectively after which time intervention is not possible [13]. Alternatively, recent methodological advances are enabling the examination of the real-time loss of ovarian follicles underlying variability in the timing of menopause, termed “ovarian aging” [14–16]. Using such methods by which the number of ovarian follicles remaining in the primordial pool (or ovarian reserve) is estimated, it is possible—for the first time—to characterize trajectories of ovarian aging over the life course. Recent work suggests, in parallel to findings in the menopausal timing literature, that even among younger, pre-menopausal women, more accelerated ovarian aging may be similarly associated with an increase in cardiovascular risk [17–21]. In this context, elucidating factors that explain variability in ovarian aging is of critical importance as it raises the possibility that such factors may be modified through intervention efforts specifically targeting the slowing of ovarian aging and/or the amelioration of its sequelae in at-risk women.

Socioeconomic status (SES) is one factor that has emerged has a reliable predictor of menopausal timing (see Gold [22]). Review of this literature shows almost one dozen studies reporting a prospective and independent effect of greater socioeconomic disadvantage on earlier menopause [23–33]. In summary, study findings suggest 1) indicators of lower SES predict earlier onset peri-menopause and menopause; 2) SES effects on earlier onset menopause are largely independent of confounding factors (e.g., smoking); and 3) the timing of lower SES exposures over time may be important. Regarding timing, studies show low SES across periods of childhood *and* adulthood conferred greatest risk, with women experiencing peri-menopause and menopause 1.2 and 1.7 years earlier, respectively, than their high SES counterparts [26, 33]. It remains unclear, however, whether it is the longer period of exposure that is important or whether there are sensitive developmental periods when exposures may be more impactful [34–39]. Notably, because the ovarian follicle pool is established in utero, exposures during this time may be especially relevant. In fact, a range of prenatal exposures (e.g., famine exposure, maternal pre-pregnancy diabetes, maternal smoking during pregnancy, multiple birth status, and both low and high birthweight) has been shown to predict earlier menopause in adult offspring [40–45] and, in the only study to examine a biomarker of ovarian aging (antimullerian hormone [AMH]), prenatal paternal smoking and maternal gestational weight gain were related to lower AMH (indexing lower ovarian reserve) while pre-pregnancy maternal history of menstrual cycle irregularity was related to higher AMH (indexing higher ovarian reserve) in adolescent offspring [46]. To date, however, no studies have examined prenatal SES-related exposures in particular.

The biological underpinnings of ovarian aging and the eventual onset of menopause reflect a complex set of processes related to 1) the initial endowment of primordial follicles occurring in utero and 2) the continuous growth of follicles beginning at the time of the initial endowment and continuing until menopause (see McGee & Hsueh [47]). Follicle growth termed “folliculogenesis” describes the progression whereby dormant primordial follicles enter the pool of growing follicles, maturing through several stages of development with the majority of follicles ultimately lost through atresia (via apoptosis) [47]. Only at puberty are a subset of these follicles rescued (via high levels of circulating follicle stimulating hormone [FSH]) with one follicle becoming dominant in preparation for the release and potential fertilization of a mature oocyte [47]. Estimates indicate that approximately 5 million follicles are present at mid-gestation, decreasing to approximately 1 million follicles at birth, 400,000 at menarche, and 10,000 at the beginning of the menopausal transition [48–50]. To date, the methodological challenges of studying ovarian follicle formation and loss have been a barrier to understanding how particular exposures may influence the size and health of the initial follicle endowment as well as the rate of ovarian follicle loss over time. As evidenced by the literatures described above, the majority of studies of prenatal exposures have been limited to the examination of ovarian aging as indexed by markers of menopausal timing [40–45], with only one study examining prenatal exposures in relation to AMH, a biochemical marker of ovarian reserve [46].

Building on the literatures described above, the current study focused on the sensitive period of follicle formation in utero by examining adverse prenatal exposures related to maternal SES. We predicted greater maternal socioeconomic disadvantage would be associated with lower ovarian reserve in the adult offspring. This hypothesis was tested by leveraging a healthy, community-based sample of reproductive age participants in the Ovarian Aging (OVA) Study (2006–2011), an investigation in which ovarian aging was assessed using well established biomarkers of total ovarian reserve, including both a biochemical marker (AMH) and an ultrasound-derived marker (antral follicle count [AFC]). Prenatal maternal SES was assessed indirectly using maternal addresses abstracted from participant birth certificates, geocoded, and linked to US Census-derived variables, including neighborhood-level education, poverty, unemployment, and income. Effects of maternal neighborhood-level SES on ovarian reserve were estimated in multivariate models adjusted for current offspring SES (educational attainment) as well as other potential confounding factors, including maternal age and offspring characteristics (age, race/ethnicity, cigarette smoking, body mass index [BMI], menarcheal age, history of hormonal contraceptive use, and parity). The current study is unique insofar as two biomarkers of ovarian reserve (AFC, AMH) were examined and that the sample itself was healthy and regularly-cycling, eliminating confounding factors that were present in prior studies.

## Methods

### Participants

Women in the current sample were participants in the Ovarian Aging (OVA) Study, a community-based investigation of reproductive aging and its correlates [18, 51–53]. Women were recruited from Kaiser Permanente of Northern California, a large, integrated healthcare delivery system that provides medical care to approximately one third of the population of Northern California. The Kaiser Permanente membership compared to the population of Northern California is generally representative in its sociodemographic and health-related characteristics, especially when the comparison is limited to those with health insurance [54]. Selection criteria for the OVA Study were age 25–45 years; regular menses; having a uterus and both ovaries intact; self-identification as white, African American, Latina, Chinese, or Filipina; and ability to speak/read English, Spanish, or Cantonese. Exclusions were major medical illnesses (i.e., cardiovascular diseases, chronic kidney or liver disease, diabetes, invasive cancer, chemotherapy or radiation therapy, epilepsy, systemic lupus erythematosus, or HIV-positive status), use of medications affecting the menstrual cycle in the 3 months prior to study participation, and current pregnancy/breastfeeding.

The OVA Study protocol included an in-person medical history interview, transvaginal ultrasound, anthropometric assessment, blood draw, and self-report questionnaires. In addition, birth certificates were obtained for a subset of women born in the state of California. Maternal addresses were abstracted from the birth certificates, geocoded, and linked to tract-level neighborhood SES variables. Of 1019 total participants, 433 women were born in California. Of these 433 women, birth certificates for 417 women were located and addresses were abstracted for 409 women. Finally, of these 409 women, geocoding to the 2010 Census tract level was successful for 350 women, leaving a final sample of 350 women available for inclusion in the current analyses. Addresses that could not be geocoded to the tract level were the result of poor quality of the address data. Institutional review board approval was obtained from Kaiser Permanente, the University of California San Francisco, and the University of Washington.

### Measures

#### Maternal neighborhood socioeconomic status (SES)

For a subset of participants in the OVA Study who were born in California, birth certificates were obtained from the California Department of Public Health Vital Records. Information on the birth certificates was abstracted, including maternal address and maternal age at the time of the participant’s birth. Maternal addresses were then geocoded to 2010 Census tracts and crosswalks were used to map 2010 Census tracts to the appropriate earlier Census—1970, 1980, 1990, 2000. Because a crosswalk was not available for 1960 Census tracts, the 1970 Census was used for women born in the 1960’s (*n* = 98). The majority of these women (82%) were born between years 1965 and 1969, supporting the use of the 1970 Census.

Census tracts were mapped to earlier censuses and census tract data were standardized using the Longitudinal Tract Database [55–57]. The LTDB uses population and area weighting to account for changes in the geographical boundaries of census tracts over time. The LTDB normalizes the census tract data from previous years to 2010 Census tract boundaries, allowing for comparison of data across censuses. Following the extraction of decennial values, linear interpolation methods were used to estimate annual values from the decennial data. SES-related variables, common to 5 US Censuses (1970, 1980, 1990, 2000, 2010), were extracted, including 1) *neighborhood-level education*: % of individuals with a high school diploma; 2) *neighborhood-level poverty*: % of families below the poverty line; 3) *neighborhood-level unemployment*: % of individuals > 16 years of age in the work force who were unemployed; and 4) *neighborhood-level income*: median family income. Prior to linear interpolation, median family income was adjusted for inflation to reflect 2010 dollars. Using the Consumer Price Index of the US Bureau of Labor Statistics, the adjustment (based on the percent change in price between indicated years) was computed by dividing the annual average Consumer Price Index for All Urban Consumers for 2010 by the annual average for the indicated earlier year.

#### Ovarian reserve

Antimullerian hormone (AMH). Blood was drawn from each study participant between menstrual cycle days 2 to 4. The concentration of AMH (ng/mL) was assayed using two commercially available enzyme-linked immunosorbent assays (ELISAs) from Beckman Coulter, both of which use a two-site sandwich immunoassay. The majority of the samples (85%) were assayed using the Immunotech assay until this assay was retired. The remainder of the samples were assayed using the second generation assay (Gen II). In a subset of 44 women in whom both assays were performed, regression analyses showed excellent correspondence between the assays (R^2^ = 0.94), which has also been demonstrated in prior studies [58, 59]. The AMH values based on the Immunotech assay were adjusted using the equation of the line with Immunotech predicting Gen II. Gen II assay sensitivity was 0.16 ng/mL, the intra-assay coefficient of variation (CV) was 1.4%, and the inter-assay CV was 12.5%.

Antral follicle count (AFC). Transvaginal ultrasound (TVUS) assessment of AFC was performed between menstrual cycle days 2 and 4 by one of two reproductive endocrinologists. The transverse, longitudinal, and anteroposterior diameters of each ovary were measured with electronic calipers using a Shimadzu SDU-450XL machine with a variable 4- to 8-mHz vaginal transducer. Follicles (defined as all echo-free structures in the ovaries) with a mean diameter across two dimensions of 2–10 mm were counted. Each measurement was taken twice and the average was taken. The total number of follicles across both ovaries was summed to calculate AFC. Evaluation of a sub-sample of 50 OVA study participants showed that inter-rater reliability between the two reproductive endocrinologists was excellent (*r* = 0.92) as was test-retest reliability for each reproductive endocrinologist measured over 2 consecutive months (average *r* = 0.91).

#### Analytical plan

Separate linear regression models were fit, examining each of four maternal neighborhood-level SES variables (education [% of individuals with a HS diploma]; poverty [% of families below the poverty line]; unemployment [% of individuals > 16 years who are unemployed]; and income [median family income]) in relation to each of two dependent measures, marking offspring ovarian reserve—AMH and AFC. In adjusted, multivariate models, all specified predictors were examined simultaneously, including all of the covariates of interest (age, maternal age, race/ethnicity, educational attainment, smoking, BMI, menarcheal age, hormonal contraceptive use, and parity) and each of the maternal neighborhood-level SES variables. The final multivariate models reflect the variables remaining after backward elimination of main effects with *P* > .10. The standardized linear regression parameters of these models are reported. Linear regression assumptions were evaluated by visual inspection and conventions for quantitative guidelines. These efforts revealed minor violations of assumptions (i.e., non-normality of residuals) that were accommodated by applying a square root transform on the positively skewed distributions of AMH and AFC.

The covariates were coded according to the following: Participant age and maternal age (abstracted from participants’ birth certificates) was coded in years. Race/ethnicity categories (white, African-American, Latina, Chinese, and Filipina) were dummy coded into four (k-1) variables using white as the reference group. Participant educational attainment categories (HS degree or less, some college, college degree, graduate degree) were dummy coded into three (k-1) variables using HS degree or less as the reference group. Cigarette smoking was coded (never smoked, current/past smoking) and BMI (kg/m^2^) was logarithmically transformed to correct positive skew. Menarcheal age was coded in years, hormonal contraceptive use was coded (no history of use, positive history of use) and parity was coded (no live births, 1+ live births). Maternal neighborhood-level SES variables were examined as continuous variables in their original units.

## Results

In Table 1, information pertaining to the sample socio-demographics characteristics, general health, ovarian reserve, reproductive factors, and maternal neighborhood-level SES is reported. The average age of the sample was 34.3 (5.6) and the average age of the participants’ mothers at the time of their births (as derived from participant birth certificates) was 26.2 (5.8). The racial/ethnic composition of the sample was 24.9% white, 43.4% African-American, 14% Latina, 13.7% Chinese, and 4.0% Filipina. This distribution differs from the total OVA Study sample (*N* = 1019; 27.4% white, 24.1% African-American, 22.6% Latina, 21.9% Chinese, and 4.0% Filipina) due to the greater number of African-American women (vs. other race/ethnic groups) who were born in the state of California and, therefore, had a birth certificate available for analysis. The sample was well-educated with 58.3% of women holding a college degree or greater, compared to 33% of women at the US population level [60]. 28.9% smoked cigarettes currently or in the past and women on average were overweight (BMI = 29.2 [7.9] kg/m^2^). Ovarian reserve indicators showed the average AMH level was 3.2 (2.6) ng/mL and the average number of antral follicles (AFC) was 15.7 (9.5). The majority of women (76%) used a hormonal form of birth control in the past and 40.6% gave birth to at least one child. Finally, examination of the neighborhoods of the participants’ mothers at the time of their births (derived from US Census data), showed the percent of individuals with a HS diploma was 66.3% on average, the percent of families living below the poverty line was 11.7% on average, the percent of individuals who were unemployed was 8% on average, and the median family income adjusted to 2010 USD was $46,497 on average.

**Table 1:** Sample characteristics (*n* = 350)

	Mean (SD)	Range	n (%)
Socio-demographics:			
Age (years)	34.3 (5.6)	25–45	–
Maternal age (years)	26.2 (5.8)	16–44	
Race/ethnicity:			
White (%)	–	–	87 (24.9)
African-American (%)	–	–	152 (43.4)
Latina (%)	–	–	49 (14.0)
Chinese (%)	–	–	48 (13.7)
Filipina (%)	–	–	14 (4.0)
Education:			
< High school (HS) (%)	–	–	7 (2.0)
HS degree (%)	–	–	38 (10.8)
Some college (%)	–	–	101 (28.9)
College degree (%)	–	–	139 (39.7)
Graduate degree (%)	–	–	65 (18.6)
General Health:			
Smoking (current/past) (%)	–	–	101 (28.9)
Body mass index (BMI) (kg/m^2^)	29.2 (7.9)	17.1–58.4	–
Ovarian Reserve:			
Antimullerian hormone (AMH)	3.2 (2.6)	0.2–13.8	–
Antral follicle count (AFC)	15.7 (9.5)	0–49	–
Reproductive Factors:			
Menarcheal age (years)	12.4 (1.7)	8–17	–
History of hormonal contraceptive use (%)	–	–	266 (76.0)
Parity (1+ live births) (%)	–	–	142 (40.6)
Maternal Neighborhood (census-tract level):			
Education: % of individuals with a HS diploma	66.3 (17.1)	20.2–98.3	–
Poverty: % of families below poverty line	11.7 (10.0)	0.6–54.8	–
Unemployment: % of unemployed individuals > 16 years	8.0 (4.3)	1.5–23.3	–
Income: Median family income (adj. to 2010 USDs)	46,497 (17,638)	13,012–110,355	–

In Table 2, bivariate correlations of unadjusted associations between maternal neighborhood characteristics and offspring ovarian reserve markers, transformed AMH and AFC, are reported. Overall, bivariate correlations suggest that greater socioeconomic disadvantage in the neighborhoods of women during pregnancy is related to lower ovarian reserve among their adult offspring. Specifically, neighborhood-level education and family income was related positively to AMH (*r* = .254, *P* < .001, *r* = .196, *P* < .001, respectively) while greater neighborhood-level poverty was related inversely to AMH (*r* = −.106, *P* < .10). Neighborhood-level education and family income was similarly related positively to AFC (*r* = .173, *P* < .001, *r* = .125, *P* < .05, respectively). As expected, associations between the maternal neighborhood characteristics (education, poverty, unemployment, income) were all significant (all P’s < .001) as was the association between AMH and AFC (*r* = .726, *P* < .001).

**Table 2:** Correlations between maternal neighborhood characteristics during pregnancy and offspring ovarian reserve in adulthood

	Maternal Neighborhood: Education	Maternal Neighborhood: Poverty	Maternal Neighborhood: Unemployment	Maternal Neighborhood: Income	AMH	AFC
Maternal Neighborhood: Education	–	−.481***	−.589***	.631***	.254***	.173**
Maternal Neighborhood: Poverty		–	.714***	−.545***	−.106†	.045
Maternal Neighborhood: Unemployment			–	−.645***	−.085	.020
Maternal Neighborhood: Income				–	196***	.125*
AMH					–	.726***
AFC						–

†*P* < .10; *P < .05; **P < .01; ***P < .001

In Table 3, results of covariate-adjusted linear regression models examining maternal neighborhood characteristics during pregnancy and offspring ovarian reserve in adulthood are reported. In the final models, following the backward elimination of main effects with *P* > .10, associations are evident between maternal neighborhood-level SES and offspring ovarian reserve. Specifically, greater maternal neighborhood education was related to *higher* ovarian reserve as marked by higher levels of offspring AMH (beta = .142, *P* < .001) and AFC (beta = .092, *P* < .10) with models accounting for 19.6% and 21.5% of the variance in AMH and AFC, respectively. Conversely, greater maternal neighborhood poverty was related to *lower* ovarian reserve as marked by lower offspring AMH (beta = −.144, *P* < .01), with the model accounting for 19.5% of the variance in AMH.

**Table 3:** Final multivariate linear regression models examining maternal neighborhood characteristics during pregnancy and offspring ovarian reserve in adulthood, adjusted for covariates.* Results show variables remaining in the models after backward elimination of main effects with *P* > .10

			DV: AMH	
	Beta	*P*	b	95% CI for b
1. Predictors:				
Age	−.318	.000	−.040	(− 0.052, − 0.028)
BMI	−.170	.001	−.472	(−0.748, − 0.196)
Maternal Neighborhood:				
Education (% of individuals with a HS diploma)	.142	.006	.588	(0.168, 1.008)
2. Predictors:				
Age	−.382	.000	−.048	(−0.061, −0.035)
BMI	−.157	.003	−.435	(−0.719, − 0.152)
Maternal Neighborhood:				
Poverty (% of families below the poverty line)	−.144	.007	−1.033	(−1.778, −0.288)
3. Predictors: Maternal Neighborhood:				
Unemployment (% of unemployed individuals)	–	n.s.	–	–
4. Predictors: Maternal Neighborhood:				
Income (median family income)	–	n.s.	–	–
			DV: AFC	
	Beta	*P*	b	95% CI for b
1. Predictors:				
Age	−.398	.000	−.086	(−0.107, −0.064)
Hormonal contraceptives	−.119	.017	−.338	(−0.616, − 0.061)
Maternal Neighborhood:				
Education (% of individuals with a HS diploma)	.092	.064	.649	(−0.038, 1.337)
2. Predictors: Maternal Neighborhood:				
Poverty (% of families below the poverty line)	–	n.s.	–	–
3. Predictors: Maternal Neighborhood:				
Unemployment (% of unemployed individuals)	–	n.s.	–	–
4. Predictors: Maternal Neighborhood:				
Income (median family income)	–	n.s.	–	–

*Covariates examined simultaneously included age (in years); maternal age (in years); race/ethnicity (using white as the reference group vs. African-American, Latina, Chinese, or Filipina); educational attainment (using HS degree or less as the reference group vs. some college, college degree, or graduate degree); smoking (0 = never smoked, 1 = current/past smoking); BMI (kg/m^2^, log transformed); menarcheal age (in years); hormonal contraceptives (0 = no history of use, 1 = positive history of use; and parity (0 = no live births, 1 = 1+ live births)

**Fig. 1:**
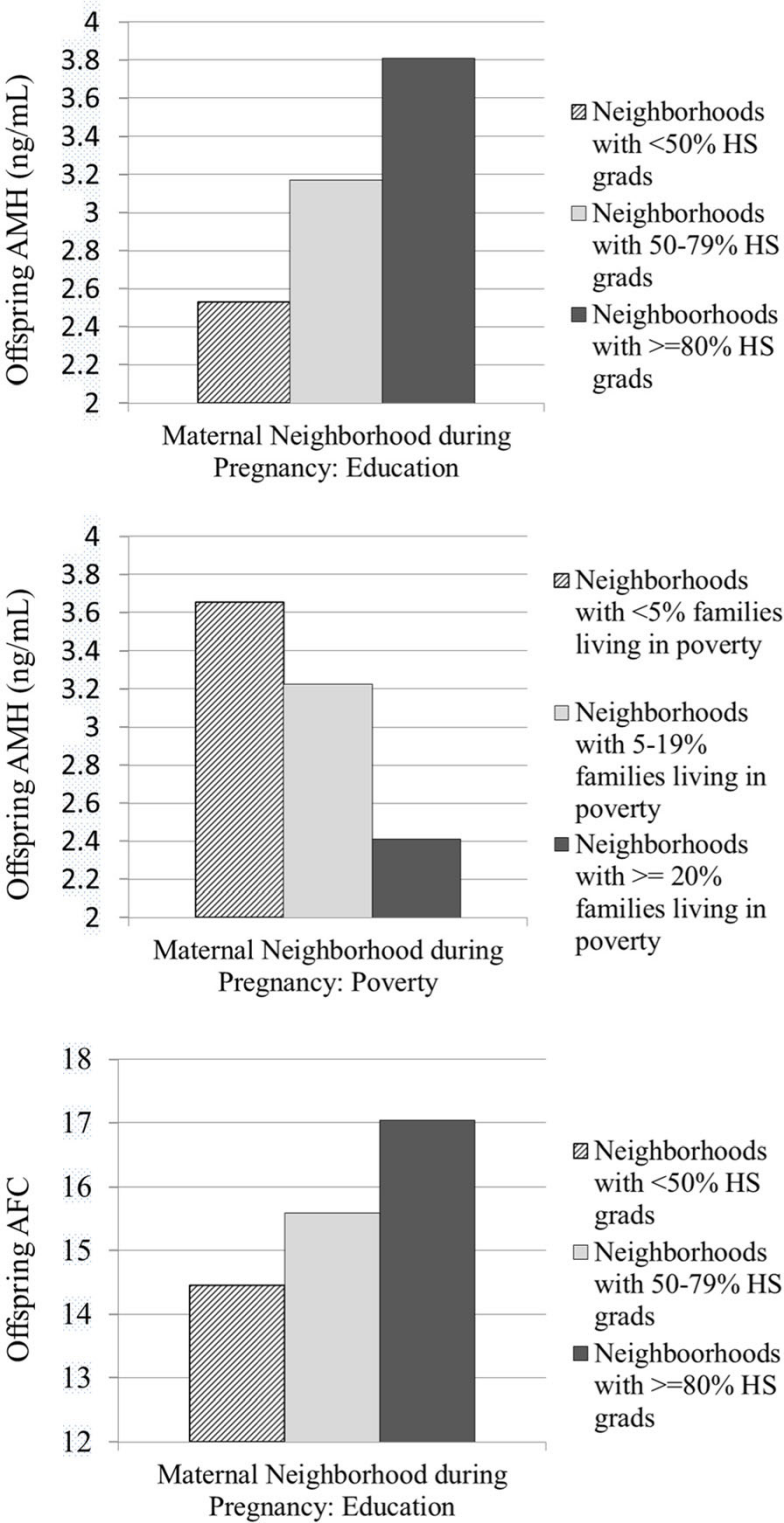
Offspring ovarian reserve markers across categories of maternal neighborhood-level SES (education, poverty), adjusted for covariates

To illustrate the significant findings in Table 3, additional linear models were fit replacing the continuous maternal neighborhood SES indicators with coarsened indicators. The effects of the categorical maternal neighborhood SES indicators are represented graphically in Fig. 1, showing ovarian reserve markers (in untransformed units) across categories of maternal neighborhood-level SES, adjusted for all covariates. Across categories of maternal neighborhood education (1 = neighborhoods with < 50% of individuals having earned a HS diploma; 2 = neighborhoods with 50–79% of individuals having earned a HS diploma; and 3 = neighborhoods with > = 80% of individuals having earned a HS diploma), adjusted marginal means for AMH were 2.5 (SE = 0.3) ng/mL, 3.2 (SE = 0.2) ng/mL, and 3.8 (SE = 0.3) ng/mL, respectively (F(2,324) = 3.6, *P* < .05). Contrasts showed significant differences between education categories 1 and 3 (*P* < .01), and marginal differences between education categories 1 and 2 (*P* < .10), and 2 and 3 (*P* < .10). Adjusted marginal means for AFC levels were 14.5 (SE = 1.2), 15.6 (SE = 0.6), and 17.0 (SE = 1.1), respectively, following a similar, albeit non-significant (F(2,323) = 1.2, *P* > .05), pattern of association. Finally, across categories of maternal neighborhood poverty (1 = neighborhoods with < 5% of families living below the poverty line; 2 = neighborhoods with 5–19% of families living below the poverty line; and 3 = neighborhoods with > = 20% of families living below the poverty line, adjusted marginal means for AMH were 3.7 (SE = 0.3) ng/mL, 3.2 (SE = 0.2) ng/mL, and 2.4 (SE = 0.3) ng/mL, respectively (F(2,324) = 3.4, *P* < .05). Contrasts showed significant differences between poverty categories 1 and 3 (*P* < .05) and 2 and 3 (*P* < .05).

## Discussion

Building on a robust literature showing lower SES is related to earlier menopausal timing [23–33], the current study focused on adverse prenatal exposures related to maternal SES, hypothesizing that greater maternal socioeconomic disadvantage would be associated with lower ovarian reserve in the adult offspring. Results supported this hypothesis. In a healthy, community-based sub-sample of reproductive age participants in the OVA Study, maternal SES measured indirectly through US Census-derived maternal neighborhood characteristics was related to ovarian reserve in the adult offspring. Specifically, greater maternal neighborhood-level education (% of individuals with a high school diploma) was related to higher offspring ovarian reserve as marked by both AMH and AFC. In addition, greater maternal neighborhood-level poverty (% of families below the poverty line) was related to lower offspring ovarian reserve as marked by AMH. These associations were present independently of offspring SES indexed by educational attainment as well as other potential confounding factors, including maternal age and offspring characteristics (age, race/ethnicity, cigarette smoking, BMI, menarcheal age, history of hormonal contraceptive use, and parity). Because the ovarian follicle pool is established in utero, the current findings are important in suggesting that SES-related adversity exposures during this period may have a detrimental impact on the size or health of the initial follicle endowment, leading to accelerated follicle loss over time.

There are several notable strengths of the current study. First, the current study implemented a novel methodological strategy to characterize SES-related adversity exposures in the prenatal period, a time period that is often neglected in the literature yet is critically important for the initial endowment of the ovarian follicle pool and subsequent trajectories of ovarian follicle loss over time, culminating at menopause. The methodological approach of the current study involved the ascertainment of objective SES-related data derived from US Censuses characterizing the neighborhoods in which the mothers of the OVA Study participants lived. Second, the current study is the first study to our knowledge to examine maternal SES, extending the current literature which has focused almost exclusively on SES in the offspring. The current study is also the first study to our knowledge to examine maternal SES in relation to established biomarkers of ovarian reserve (AMH, AFC), extending the current literature which has focused almost exclusively on menopausal timing. The inclusion of these biomarkers offers a unique opportunity to examine prenatal exposures in relation to variability in ovarian aging among younger women when fertility preservation may still be possible. Although there are prior studies of prenatal adversity exposures, none have considered SES exposures in particular [40–45] and only one examined a biomarker of ovarian reserve [46]. Lastly, the current study drew from a large, well-characterized group of reproductive age participants in the OVA Study. These women were healthy, regularly cycling, and not taking hormonal contraceptives, eliminating numerous potential confounds, including the inclusion of women with polycystic ovarian syndrome (PCOS).

There are several notable weaknesses of the current study. First, maternal neighborhood-level SES is only an indirect marker of individual-level maternal SES. It is possible that high SES mothers may live in lower SES neighborhoods and/or be able to avoid exposures associated with low SES environments. In this way, use of a neighborhood-level marker may not be an adequate representation of an individual mother’s experiences. The current study did not have direct measures of maternal SES such as educational attainment, income level, or wealth. Second, although the current study attempted to isolate the prenatal period, realistically, it is not possible to discern the impact of exposures related to the prenatal period versus the postnatal period. In fact, it is likely that the neighborhood-level SES exposures present prenatally persisted into infancy and childhood may have continued to exert a deleterious influence on the offspring ovarian reserve. In this way, pointing to initial follicle endowment as the specific process that may have been disrupted remains speculative, although it is noteworthy that the effects of maternal neighborhood-level SES on offspring ovarian reserve were independent of offspring SES. Lastly, the current study did not have measures that might help elucidate *why* maternal neighborhood-level SES may impact offspring ovarian reserve. The current study did not have direct assessments of relevant variables such as maternal health behaviors, nutritional status, and general health as well as potentially correlated exposures such as environmental toxicants known to be endocrine-disrupting.

Future research should improve upon the weaknesses of the current study by focusing on particular maternal and environmental factors that might be driving observed associations between maternal neighborhood-level SES and offspring ovarian reserve. Prior study findings highlight more broadly the impact of early life environments on subsequent reproductive health outcomes. For example, using a novel study design, women who grew up in Sylhet, Bangladesh were shown to have hormonal profiles consistent with lower ovarian reserve and reduced fertility compared to their Bangladeshi counterparts who migrated to Britain as children (versus as adults) as well as to other European-born women who grew up in Britain [61, 62]. This suggests that early life adversity exposures in Bangladeshi neighborhoods, possibly reflecting exposures to nutritional stress, infectious disease, or other yet unidentified stressors, may detrimentally impact adulthood reproductive health outcomes. In animal models, experimental studies have focused on poor maternal nutritional status in particular, showing maternal undernutrition and malnutrition were related to markers of impaired folliculogenesis, lower ovarian reserve, and increased oxidative stress in the adult offspring [63–67]. Consistent with these findings, in a prior study of women, maternal nutritional deprivation during famine was associated with earlier menopausal timing in the offspring [44] as was pre-pregnancy diabetes [43].

In parallel, other studies have investigated the relevance of maternal smoking to offspring reproductive health. In animal models, experimental studies showed that exposure to maternal smoking, similar to study findings regarding maternal nutritional status, was related to negative reproductive health outcomes in the adult offspring, including indicators of sub-fertility, lower ovarian reserve, and increased oxidative stress [68–70]. Consistent with these findings, in a prior study of women, maternal smoking during pregnancy was associated with earlier menopausal timing in the offspring [41]. These authors suggested that maternal smoking might influence the hormonal environment in utero in a way that negatively impacts the formation of the ovarian reserve and subsequent follicle loss. In fact, lower estradiol and estriol levels have been documented in pregnant smokers [71–74]. Because estradiol appears to play a role in the maintenance of the primordial follicle pool [75], lower estradiol levels associated with maternal smoking may allow premature follicle growth, hastening the depletion of the ovarian reserve.

Taken together, mounting epidemiological and experimental evidence suggests that variation in adulthood ovarian function has developmental origins in the intrauterine and early childhood environments, suggesting particular exposures during these sensitive periods may shape trajectories of ovarian aging. Exposures related to maternal nutritional stress and maternal smoking, which are also significantly correlated with lower SES [76–78], are strong candidates for inclusion in future research to address whether maternal neighborhood-level SES in the current study may be marking behavioral, anthropometric, or other health-related characteristics of the mother.

In addition, although less strongly supported, it is possible that maternal neighborhood-level SES may be marking toxicant exposures that cluster in low SES environments [79–81]. A particular group of chemical exposures known to interfere with the actions of hormones termed “endocrine-disrupting chemicals (EDCs)” [82, 83] are common in personal care and household products with recent evidence documenting elevations in particular EDCs (e.g., lead, cadmium, bisphenol A [BPA]) among lower SES individuals [84]. Exposures to EDCs have been related to a host of reproductive health outcomes, including earlier onset puberty, infertility, endometriosis, PCOS, uterine fibroids, and pregnancy complications [85–94]. With respect to ovarian aging outcomes in particular, a recent review [95] summarized relevant human and animal literatures, suggesting environmental toxicants accelerate folliculogenesis and follicular atresia, including in the primordial stage and extending across the spectrum of ovarian follicle development. Notably, in a large representative sample of US women (*n* = 31,575) EDCs (i.e., polychlorinated biphenyl [PCBs], pesticides, furans, and phthalates) were associated with earlier onset menopause up to 3.8 years earlier, following adjustment for covariates [96]. In addition, prospective studies showed higher urinary phthalate and BPA levels were related to decreases in ovarian reserve as marked by lower AFC, although participants were patients seeking infertility treatment [97, 98]. To our knowledge, no studies have examined EDCs in relation to biomarkers of ovarian reserve (AFC, AMH) in healthy, reproductive age women.

## Conclusions

In conclusion, results from the current study showed maternal neighborhood-level SES was related to offspring ovarian reserve, independently of a host of confounding variables, including offspring SES. These findings suggest that in the prenatal period, adverse exposures related to increased maternal socioeconomic disadvantage may have a detrimental impact on offspring ovarian aging possibly via disruptions in the initial follicle endowment. Future work, however, is necessary to elucidate the mechanisms that may explain this association, including whether specific maternal (e.g., health behaviors) or environmental (e.g., EDCs) factors that are commonly correlated with neighborhood-level SES may be driving these associations. Future work should also be guided by a focus on the timing and time course of exposures as well as the dual consideration of both maternal and offspring characteristics. The clinical implications of these results are that risk factors for accelerated ovarian aging in offspring may be identified in mothers prenatally. Insofar as such risk factors are able to be modified prenatally or even before conception, this work offers novel directions for potential interventions to improve the health of mothers and their environments, thereby maximizing the long-term reproductive health of their offspring. Moreover, as evidence mounts that reproductive health and aging are related more broadly to cardiovascular risk [17–21], the implications of this work for the general health and well-being of women are far-reaching.

## Abbreviations

AFC: Antral follicle count
AMH: Antimullerian hormone
BMI: Body mass index
BPA: Bisphenol A
EDC: Endocrine-disrupting chemical
ELISA: Enzyme-linked immunosorbent assay
FSH: Follicle stimulating hormone
LTDB: Longitudinal Tract Database
OVA Study: Ovarian Aging Study
PCB: Polychlorinated biphenyl
PCOS: Polycystic ovarian syndrome
SES: Socioeconomic status
